# Evaluation efficacy and accuracy of a real-time computer-aided polyp detection system during colonoscopy: a prospective, multicentric, randomized, parallel-controlled study trial

**DOI:** 10.1007/s00464-025-12080-x

**Published:** 2025-09-02

**Authors:** Xin Xu, Ling Ba, Lin Lin, Yan Song, Chunshan Zhao, Shuangzhe Yao, Hailong Cao, Xin Chen, Jinbao Mu, Lu Yang, Yue Feng, Yufeng Wang, Bangmao Wang, Zhongqing Zheng

**Affiliations:** 1 Department of Gastroenterology and Hepatology, Tianjin Medical University General Hospital, Anshan Road No.154, Tianjin, 300052 China; 2 Tianjin Yujin Artificial Intelligence Medical Technology Co.,Ltd, Tianjin, China

**Keywords:** Computer-aided detection system, Colonoscopy, Artificial intelligence, Sensitivity, Specificity, Polyp detection rate

## Abstract

**Introduction:**

Colorectal cancer (CRC) ranks as the second deadliest cancer globally, impacting patients’ quality of life. Colonoscopy is the primary screening method for detecting adenomas and polyps, crucial for reducing long-term CRC risk, but it misses about 30% of cases. Efforts to improve detection rates include using AI to enhance colonoscopy. This study assesses the effectiveness and accuracy of a real-time AI-assisted polyp detection system during colonoscopy.

**Materials and methods:**

The study included 390 patients aged 40 to 75 undergoing colonoscopies for either colorectal cancer screening (risk score ≥ 4) or clinical diagnosis. Participants were randomly assigned to an experimental group using software-assisted diagnosis or a control group with physician diagnosis. The software, a medical image processing tool with B/S and MVC architecture, operates on Windows 10 (64-bit) and supports real-time image handling and lesion identification via HDMI, SDI, AV, and DVI outputs from endoscopy devices. Expert evaluations of retrospective video lesions served as the gold standard. Efficacy was assessed by polyp per colonoscopy (PPC), adenoma per colonoscopy (APC), adenoma detection rate (ADR), and polyp detection rate (PDR), while accuracy was measured using sensitivity and specificity against the gold standard.

**Results:**

In this multicenter, randomized controlled trial, computer-aided detection (CADe) significantly improved polyp detection rates (PDR), achieving 67.18% in the CADe group versus 56.92% in the control group. The CADe group identified more polyps, especially those 5 mm or smaller (61.03% vs. 56.92%). In addition, the CADe group demonstrated higher specificity (98.44%) and sensitivity (95.19%) in the FAS dataset, and improved sensitivity (95.82% vs. 77.53%) in the PPS dataset, with both groups maintaining 100% specificity. These results suggest that the AI-assisted system enhances PDR accuracy.

**Conclusion:**

This real-time computer-aided polyp detection system enhances efficacy by boosting adenoma and polyp detection rates, while also achieving high accuracy with excellent sensitivity and specificity.

Colorectal cancer (CRC) ranks as the third most commonly diagnosed malignancy and the second leading cause of cancer-related mortality [1]. The onset and progression of colorectal cancer involve highly complex processes, with numerous factors contributing to mortality [2–4]. Research has indicated an association between CRC and various diseases, including inflammatory bowel diseases (IBDs), complicating treatment efforts and hindering the achievement of a favorable prognosis [2]. Despite the identification of numerous therapies for colorectal cancer, the recovery rates and prognoses have not met expectations [5–7]. Given that the disease typically manifests symptoms only at advanced stages, pre-cancer screening is evidently crucial in enhancing early detection and reducing both morbidity and mortality associated with colorectal cancer [8]. Consequently, emphasis should be placed on screening strategies to prevent colorectal cancer; implementing effective colorectal cancer (CRC) screening programs can significantly mitigate the increasing burden of the disease [9, 10].

Recent studies have established a clear association between the reduction of colorectal cancer risk and the implementation of screening colonoscopy [11]. Colonoscopy is essential for both the diagnosis and treatment of lower gastrointestinal disorders [12]. As the gold standard for diagnosing colorectal cancer, colonoscopy allows for comprehensive visualization of the entire lower gastrointestinal tract and facilitates the removal of lesions. However, the primary cause of interval colorectal cancer is often attributed to missed diagnoses during colonoscopy, with the failure to recognize polyps being a significant factor contributing to this miss rate of colorectal neoplasia [13, 14]. Furthermore, research has indicated that the effectiveness of screening colonoscopy is somewhat limited, highlighting the need for broader implementation and adoption of effective colorectal cancer screening strategies [15].

Importantly, the integration of artificial intelligence (AI)—defined as machines or software systems capable of performing tasks typically associated with human intelligence—into the medical field has shown promise in enhancing patient outcomes across various domains [16, 17]. Furthermore, artificial intelligence has been utilized to aid in the early diagnosis of colorectal cancer during colonoscopy by enhancing the detection of adenomas and polyps [18, 19]. Research has demonstrated that the detection of adenomas and polyps, which are pathological indicators associated with precancerous lesions of colorectal cancer, is inversely related to the risk of interval colorectal cancer, advanced-stage interval cancer, and fatal interval cancer [20–23]. Consequently, there has been a growing application of artificial intelligence to improve the detection of adenomas and polyps for early-stage colorectal cancer screening, a practice that has been ongoing for several years [24, 25]. For instance, the accuracy of polyp diagnosis has been enhanced through the use of convolutional neural networks with visual explanations [25]. Notably, compared to traditional colonoscopy performed by endoscopists, artificial intelligence-assisted colonoscopy (AIC) is a safe and highly effective screening tool that can increase the detection rate of adenomas and polyps, thereby facilitating the early diagnosis of colorectal cancer [26].

Previous research has established that computer-aided detection (CADe) significantly enhances the efficacy of colonoscopic polyp and adenoma detection [27]. Nonetheless, some studies have indicated that CADe is associated with a high false-positive rate [28]. A limited number of articles have systematically reviewed and assessed the accuracy of CADe-assisted colonoscopic polyp detection. Consequently, to further evaluate the accuracy of CADe-assisted colonoscopic polyp detection, we conducted the present clinical trial. Moreover, our prior study preliminarily demonstrated that the application of a software marker significantly assisted endoscopists in identifying a greater number of polyps across 85 video recordings of senior colonoscopy examinations [29]. In this prospective, multicenter, randomized, parallel-controlled study, we aimed to assess the efficacy of this real-time computer-aided polyp detection system in comparison to routine diagnostic practices employed by physicians. Furthermore, we sought to evaluate the accuracy of the software’s independent detection of polyps and adenomas relative to standard diagnostic procedures.

## Materials and methods

### Study design

We conducted a prospective, multicenter, randomized study to validate the efficacy and safety of a real-time computer-aided polyp detection system during colonoscopy. We compared the software’s sensitivity in identifying polyps and adenomas and its specificity in distinguishing non-polyp tissue. The trial was conducted at eight hospitals: Tianjin Medical University General Hospital, Beijing Friendship Hospital, Cangzhou Central Hospital, Taiyuan Central Hospital, The Second Hospital of Hebei Medical University, The Eighth Affiliated Hospital of Sun Yat-sen University in Shenzhen, and Beijing Hospital. The software’s image processing system is approved as a Class II device by the Tianjin Municipal Market and Quality Supervision Administration Committee, allowing for clinical trials. This experiment requires adherence to NMPA guidelines for AI-assisted detection software and compliance with quality management standards for clinical trials of medical devices.

## Participants

The study included patients aged 40 to 75 scheduled for colonoscopy due to colorectal cancer screening with a risk score of ≥ 4 or clinical needs. Exclusions were made for those with a polypectomy in the past 5 years, history of abdominal/pelvic surgery, radiotherapy, chemotherapy, active intestinal bleeding, hereditary colorectal polyposis, inflammatory bowel disease, uncontrolled hypertension, history of stroke, coronary artery disease, inability to prepare intestines, conditions preventing endoscopy, recent participation in other trials, or any other factors deemed unsuitable by researchers.

### Algorithm

**Fig. 1:**
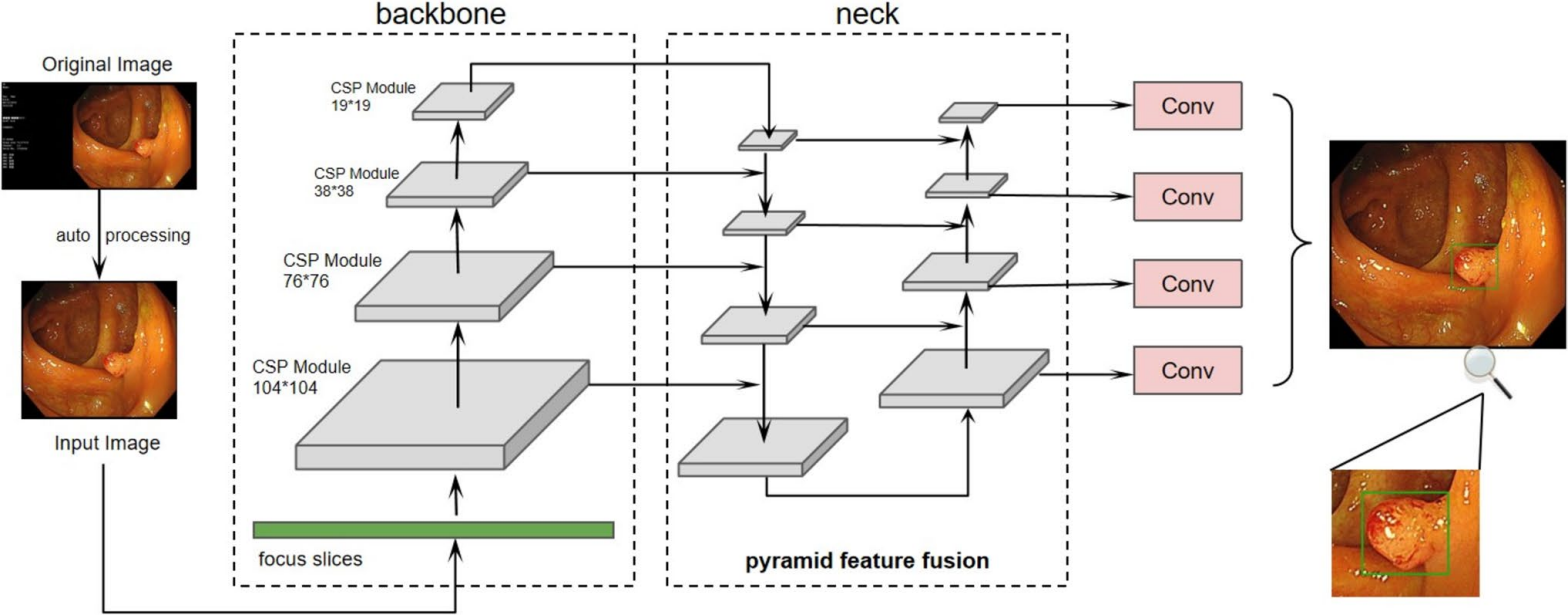
Architecture diagram of assisted diagnosis software

In this study, we advanced beyond our previous methodologies by employing the YOLOv5l deep learning model to develop a system for colorectal polyp detection [27]. The YOLOv5l model, a single-stage object detection algorithm, excels in accuracy and real-time performance, making it ideal for medical image analysis. It uses a CSP backbone, SPP module, and PANet structure to detect polyps of various sizes and shapes. The training dataset comprised 17,277 positive samples (from proliferative polyps to adenocarcinoma) and 5,591 negative samples (normal intestine, light spots, bubbles, feces). A gastroenterology specialist annotated the images, with a senior expert reviewing for quality. Preprocessing involved cropping, enhancing (via rotation, flipping, brightness, and contrast adjustments), and normalizing images. The model was trained using transfer learning, starting with COCO dataset pre-trained weights and fine-tuning on our polyp dataset. The Adam optimizer was used with an initial learning rate of 0.001, employing a cosine annealing strategy. Early stopping and weight decay were applied to prevent overfitting. The dataset was split into training, validation, and test sets in a 7:2:1 ratio. The training set is used for algorithm learning, the validation set for initial validation and optimization, and the test set evaluates the algorithm's performance on new data, achieving a sensitivity of 97.18%. The CADe system employed in this study performs frame-by-frame image prediction of endoscopic signals using a target detection algorithm based on YOLOv5 [30]. This system marks the bounding box of suspicious polyps in real time, with a delay time of (16 ± 4) ms. The underlying principle of this network involves extracting features from color frames via the Backbone portion of the network to generate multi-scale feature maps. Subsequently, these feature maps are fused and enhanced through a multi-level feature pyramid structure. Finally, positional regression is applied to infer the boundary range of the region of interest (Fig. 1). In addition, the system incorporates a pre-discrimination module that displays detection results persisting for more than two consecutive frames at the same recognized location in the prediction output, thereby initially reducing false-positive alarms. Developed using 22,868 images, the algorithm achieves a sensitivity of 97.18% and an accuracy of 92.83% on the test set, demonstrating particularly strong performance in detecting flat micro-polyps [31, 32].

## Randomization

The clinical trial center screened eligible subjects and randomly assigned them to either the experimental or control group before their colonoscopy. In the experimental group, colonoscopies were performed with software assistance, while the control group underwent routine procedures. Block randomization was used, with random numbers generated by SAS ®9.4 software to ensure a 1:1 ratio between groups. Individuals not involved in the trial prepared the random grouping cards, and neither researchers nor subjects were informed of group assignments to prevent selection bias. Due to the software-assisted intervention, blinding was not possible for patients and researchers. No control medical devices were used in the study.

The trial will be conducted across multiple clinical institutions, aiming for an even distribution of centers to ensure central representation. However, enrollment will depend on feasibility and selection progress, with adjustments made to maintain balance. No single center will exceed 50% of the total cases.

## Procedure

**Fig. 2:**
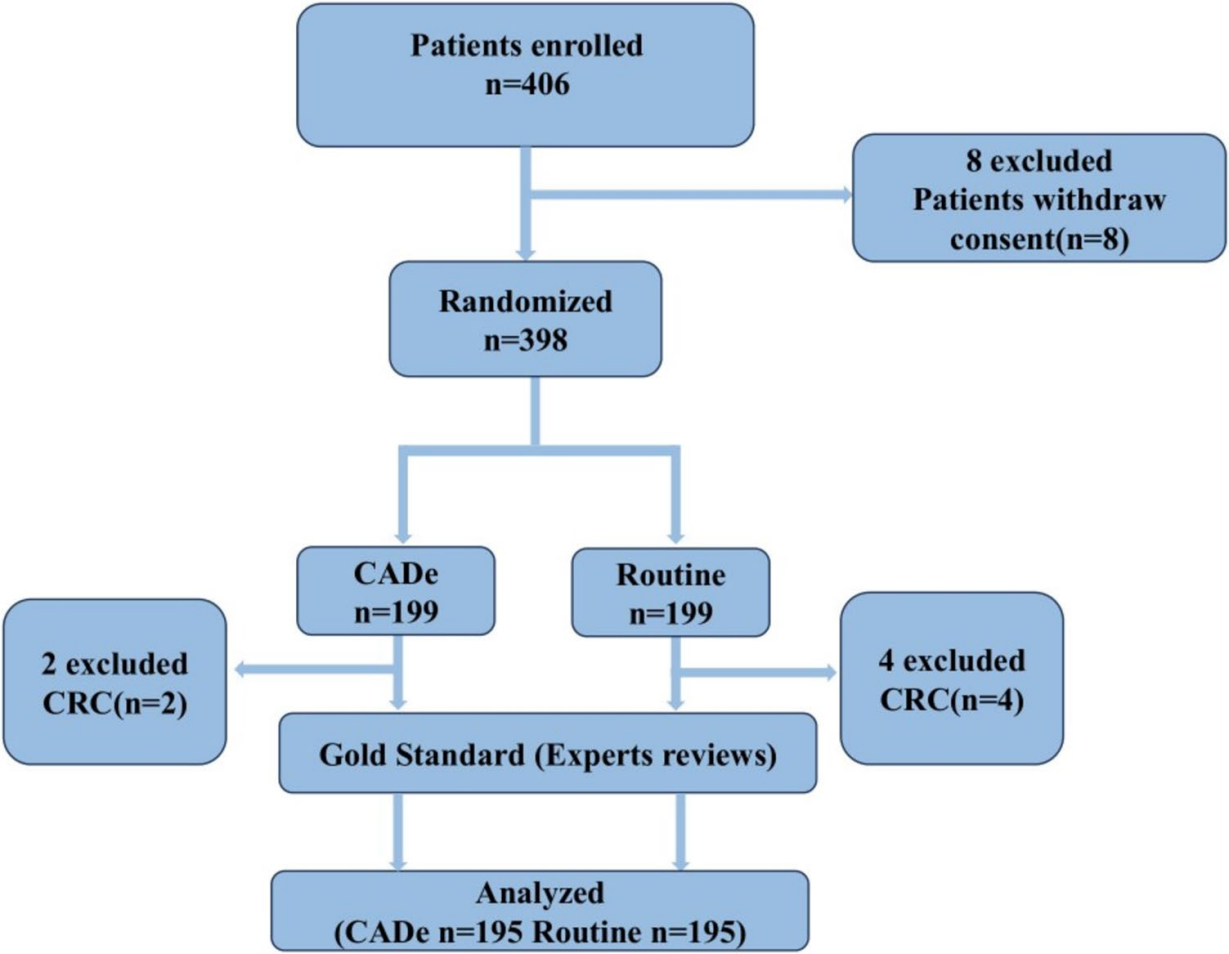
Flowchart of experimental process

Participants meeting the inclusion criteria joined the study for about 7 days, during which they provided informed consent, and their basic information was collected. The included patients were randomly divided into two groups. One group was performed by the physician for routine colonoscopy using only colonoscopy equipment; this group was called control group called standard colonoscopy group. In the other group, the physician performed the colonoscopy using the colonoscopy device with an assisted detection software, which was called the experimental group, the AI-assisted colonoscopy group. The AI-assisted detection software only served as a prompt, it marked the suspected polyps for further judgment by the physician (Fig. 2). The experimental group’s endoscopist used a workstation screen to observe lesions and record polyp numbers. Lesion location, number, and size were documented for both groups. Pathologic tissue was also taken and sent for examination. The project team later gathered the endoscopy and pathology results as required. Adverse events including bleeding and perforation did not occur in either colonoscopy group. Pathology can only be used as the gold standard for the histologic nature of polyps, and expert judgment can determine whether a polyp is a polyp and the size of the polyp; therefore, for the sake of determining the sensitivity and specificity of the software, we have created another gold standard of expert judgment. A third-party evaluation team, led by the group leader, assessed endoscopy videos for central polyps. Two experienced gastroenterologists (with at least 15 years of experience) evaluated the videos to establish a gold standard. If they disagreed, a third gastroenterologist (with at least 20 years of experience) arbitrated. Monitoring was conducted at various stages of the clinical trial.

## Outcome measures

The primary endpoint analyzed was the sensitivity and specificity of polyp recognition between the test and control groups. Secondary endpoints included the average number of polyps per colonoscopy (PPC), adenomas per colonoscopy (APC), adenoma detection rate (ADR), and polyp detection rate (PDR), compared between the experimental and control groups. Furthermore, the sensitivity and specificity of polyp recognition were evaluated as secondary endpoints in both the software-aided group and the control group, in comparison to the gold standard.

We evaluated polyp detection sensitivity in both experimental and control groups using the gold standard. We recorded lesion locations, total polyp count, and size, aligning these to assess consistency between groups and the gold standard. A positive result was when both groups and the gold standard identified a polyp at a lesion. Sensitivity was the ratio of true positives to total positives. A negative result meant no polyps per the gold standard, with true negatives when all agreed on non-polyps. Specificity was the ratio of true negatives to total negatives. PPC indicated the average polyps per patient during colonoscopies. APC was the number of colonoscopies confirming adenomas via histopathology. ADR was APC divided by total colonoscopies. PDR measures the rate of colonoscopies that detect at least one polyp, excluding post-polypectomy monitoring, relative to the total number of colonoscopies, to evaluate efficacy. We compare these metrics between a control group and software diagnosis. A positive result matches the gold standard at the lesion level, with TP when both the control/software and gold standard identify a polyp. Sensitivity is calculated as TP divided by positives. A negative result means no polyps, with TN when both the control/software and gold standard agree. Specificity is TN divided by negatives. We also evaluate software safety by comparing bleeding and perforation rates between experimental and control groups.

## Statistical analysis

The trial will enroll 390 patients to test hypotheses and diagnostic performance using two datasets: the full analysis set (FAS) for all randomized subjects undergoing endoscopy, and the per-protocol set (PPS) for those completing the trial without major protocol breaches. Main efficacy will be evaluated with both datasets, while baseline, secondary efficacy, and safety will rely on the FAS. Count data will be shown as frequency and proportion, and measurement data will include mean, standard deviation, and percentiles. Inter-group comparisons will use the likelihood ratio Chi-square test or Fisher’s exact test for count data, and the independent samples t-test or Wilcoxon rank-sum test for measurement data. The primary endpoint is analyzed with the CMH Chi-square test, adjusted for center effects. Sensitivity and specificity for identifying polyps are estimated for both groups, with differences and 95% confidence intervals. The lower confidence limit is compared to a preset boundary for clinical applicability. Other efficacy indicators are analyzed like the baseline. Intra-group comparisons will utilize paired t-tests for normal data, Wilcoxon signed-rank tests for non-normal data, and McNemar’s Chi-square tests for qualitative data. Lab results will indicate the number and proportion of cases shifting from normal to abnormal in both groups. Adverse events will be reported by occurrence and incidence rate, analyzed using likelihood ratio Chi-square or Fisher’s exact test, and described by manifestation, severity, and relation to the study product. Device defects will be reported by incidence rate.

The primary indexes will be analyzed with a one-sided significance level of 0.025, while other indicators will use a two-sided level of 0.05 unless specified otherwise. Analysis will be conducted using SAS® 9.4. Missing data for the primary indexes will be handled with carry-over methods like the Worst Case Carry Forward, as outlined in the statistical analysis plan. Other indexes will use observed data without carry-forward methods. Abnormal data will be corrected during data cleaning before analysis.

## Results

### The baseline characteristics of patients

A total of 390 eligible subjects from eight hospitals were included in the study and randomly divided into two equal groups of 195: an experimental group and a control group. Analysis showed no significant differences in baseline characteristics such as sex, age, smoking history, family history of colorectal cancer, colonoscopy indications, cancer risk score, procedure timing, and comorbidities. However, slight statistical differences were noted in BMI, alcohol consumption, colonoscopy indication for cancer screening, and cancer risk score, with a p-value under 0.05 (Table 1).

**Table 1:** Baseline characteristics

	Computer-aided colonoscopy (*n* = 195)	Standard colonoscopy (*n* = 195)	*P*-value
*Sex, n (%)*			
Male	118(60.51)	114(58.46)	0.599
Female	77(39.49)	81(41.54)	0.524
Mean age (SD), years	56.25(9.03)	56.25(9.03)	1.000
*Age category, years, n (%)*			
40–54	86(44.10)	86(44.10)	1.000
55–64	67(34.36)	67(34.36)	1.000
65–75	42(21.54)	42(21.54)	1.000
*BMI, kg/m*^*2*^*, n (%)*			
BMI < 23	58(29.74)	77(39.48)	0.009
BMI ≥ 23	137(70.26)	118(60.52)	0.019
Smoking history, n (%)	74(37.95)	65(33.33)	0.096
Drinking history, n (%)	32(16.41)	43(22.05)	0.024
Family history of colorectal cancer in first-degree relatives, n (%)	12(6.15)	9(4.62)	0.186
*Indication for colonoscopy, n (%)*			
CRC screening	54(27.69)	67(34.36)	0.037
GI symptoms	132(67.69)	117(60.00)	0.051
FIT^+^	2(1.03)	1(0.51)	0.223
Surveillance	7(3.59)	10(5.13)	0.140
*Risk score of colorectal cancer, n (%)*			
< 4	152(77.95)	165(84.62)	0.095
≥ 4	43(22.05)	30(15.38)	0.007
*Time of colonoscopy procedure, n(%)*			
Morning	80(41.03)	83(42.56)	0.638
Afternoon	115(58.97)	112(57.44)	0.707
Comorbidity, n (%)			
Diabetes	10(5.13)	8(4.10)	0.342
Coronary heart disease	0(0.00)	2(1.03)	0.157

*SD*, standard deviation; *CRC*, colorectal cancer; *GI*, gastrointestinal; *FIT*, fecal immunochemical test

## Characteristics of polyps

In comparing polyp characteristics between two groups, we found that the CADe group significantly improved detection rates for polyps of all kinds of sizes, indicating the software’s effectiveness in identifying lesions. In addition, it significantly enhanced the detection of flat-shaped (p = 0.016) and inflammatory polyps (p < 0.001) compared to standard colonoscopy (Table 2).

**Table 2:** Characteristics of polyps

	Computer-aided colonoscopy (*n* = 195)	Standard colonoscopy (*n* = 195)	P-value
*Location, n (%)*			
Proximal colon	76(38.97)	81(41.54)	0.429
Distal colon	119(61.03)	114(58.46)	0.512
*Size category, n (%)*			
≤ 5 mm	457(80.88)	312(80.62)	< 0.001
> 5 mm	108(19.12)	75(19.38)	0.015
*Shape, n (%)*			
Pedunculated	109(33.03)	97(34.28)	0.088
Sessile	67(20.30)	56(19.79)	0.056
Flat	131(39.70)	112(39.58)	0.016
*Pathology, n (%)*			
Carcinoma	128(25.10)	116(34.22)	0.100
Inflammatory polyp	382(74.90)	223(65.78)	< 0.001

## Colonoscopy procedures

The trial indicates no significant difference in polyp detection between withdrawal and insertion times, and anesthesia does not affect detection rates. However, inadequate bowel preparations significantly differ between the CADe and control groups (*p* = 0.001, Table 3). Missed polyp detections are significantly influenced by polyp characteristics, especially unclear boundaries (*p* = 0.021), and insufficient air inflation during colonoscopy significantly affects the polyp detection rate (*p* = 0.021, Table 4).

**Table 3:** Characteristics of colonoscopy procedures

	Computer-aided colonoscopy (*n* = 195)	Standard colonoscopy (*n* = 195)	P-value
Withdrawal mean time (min)	26	28	0.586
Withdrawal mean time, excluding biopsy time (min)	7	8	0.604
Insertion mean time (min)	5	6	0.545
No polyp withdrawal mean time (min)	26	30	0.298
*Anesthesia, n (%)*			
Yes	113(57.95)	106(54.36)	0.355
No	82(42.05)	89(45.64)	0.295
Boston score, mean (SD)	6.85(0.53)	6.93(0.37)	0.081
*Boston score, n (%)*			
Inadequate (Sum < 6.0 or anyone < 2.0)	15(7.69)	7(3.59)	0.001
Adequate (Sum ≥ 6.0 and everyone ≥ 2.0)	180(92.31)	188(96.41)	0.402

**Table 4:** Characteristics of polyps missed at endoscopy among patients in computer-aided detection group

	Computer-aided colonoscopy (*n* = 195)	Standard colonoscopy (*n* = 195)	P-value
*Polyp characteristics, n (%)*			
Isochromatic	2(1.03)	1(0.51)	0.223
Flat	18(9.23)	22(11.28)	0.083
Unclear boundary	1(0.51)	3(1.54)	0.021
Partly behind colon folds	7(3.59)	9(4.62)	0.312
On the edge of visual field	6(3.08)	5(2.56)	0.545
*Colon condition, n (%)*			
Insufficient air inflation	3(1.54)	1(0.51)	0.021
Part occlusion by liquid feces or debris	2(1.03)	2(1.03)	1.000
*Endoscopist, n (%)*			
Withdrew equipment too fast	4(2.05)	3(1.54)	0.445
Distracted	0(0.00)	0(0.00)	1.000
*Endoscopy, n (%)*			
Overexposure	2(1.03)	1(0.51)	0.223
Blurred lens	0(0.00)	0(0.00)	1.000
Insufficient light	0(0.00)	0(0.00)	1.000

## Indexes of the trial

The ADR was significantly higher in the CADe group (40.00%) compared to the control group (39.49%). Similarly, the PDR was elevated in the CADe group (67.18%) relative to the control group (56.92%). In addition, the CADe group identified a greater number of polyps per colonoscopy, particularly those measuring 5 mm or smaller (61.03% in the CADe group versus 56.92% in the control group, as shown in Table 5. The CADe group also demonstrated improved specificity (98.44%) and sensitivity (95.19%) in the full analysis set (FAS), as well as enhanced sensitivity (95.82% compared to 77.53%) in the per-protocol set (PPS), with both groups exhibiting 100% specificity. These results suggest that the AI-assisted system significantly enhances the accuracy of PDR (see Table 6).

**Table 5:** Secondary indexes of the trial

Index	Computer-aided colonoscopy (*n* = 195)	Standard colonoscopy (*n* = 195)	P-value
PPC (95% CI)	2.91 (2.83, 2.99)	2.01 (1.93, 2.09)	< 0.001
≤ 5 mm	2.36 (1.45, 3.27)	1.62 (0.89, 2.35)	0.430
> 5 mm	0.56 (0.27, 0.85)	0.39 (0.17, 0.61)	0.400
APC (95% CI)	1.04 (0.96, 1.12)	1.12 (1.04, 1.20)	0.023
≤ 5 mm	0.68 (0.38, 0.98)	0.79 (0.49, 1.09)	0.560
> 5 mm	0.36 (0.17, 0.55)	0.33 (0.14, 0.52)	0.630
ADR (95% CI)	40.00% (33.5%, 46.5%)	39.49% (33.0%, 45.9%)	0.888
≤ 5 mm	29.74% (24.07%, 35.41%)	31.79% (26.11%, 37.47%)	0.380
> 5 mm	14.87% (10.72%, 19.02%)	12.82% (9.31%, 16.33%)	0.200
PDR (95% CI)	67.18% (60.9%, 73.4%)	56.92% (50.3%, 63.5%)	< 0.001
≤ 5 mm	61.03% (55.12%, 66.94%)	51.28% (45.69%, 56.87%)	0.0008
> 5 mm	26.15% (20.79%, 31.51%)	22.56% (17.65%, 27.47%)	0.141

*PPC*, average number of polyps per colonoscopy; *APC*, adenoma per colonoscopy; *ADR*, adenoma detection rate; *PDR*, polyp detection rate

**Table 6:** Primary indexes of the trial

Index	Computer-aided colonoscopy (n = 195)	Standard colonoscopy (n = 195)	P-value
*FAS*			
TP (95% CI)	563 (0.947, 0.969)	376 (0.736, 0.816)	< 0.001
P (95% CI)	24 (0.031, 0.053)	109 (0.184, 0.264)	< 0.001
TN (95% CI)	63 (0.968, 0.999)	71 (0.956, 0.988)	0.345
N (95% CI)	1 (0.001, 0.031)	2 (0.010, 0.046)	0.625
Sensitivity (95% CI)	95.91% (93.9%, 97.9%)	77.53% (74.5%, 80.5%)	< 0.001
Specificity (95% CI)	98.44% (97.4%, 99.5%)	97.26% (96.3%, 98.2%)	0.070
*PPS*			
TP (95% CI)	550 (0.945, 0.971)	376 (0.735, 0.815)	< 0.001
P (95% CI)	24 (0.028, 0.056)	109 (0.185, 0.265)	< 0.001
TN (95% CI)	62 (1.00, 1.00)	69 (1.00, 1.00)	1.00
N (95% CI)	0 (0.00, 0.00)	0 (0.00, 0.00)	1.00
Sensitivity (95% CI)	95.82% (93.8%, 97.8%)	77.53% (74.5%, 80.5%)	< 0.001
Specificity (95% CI)	100.00% (100.00%, 100.00%)	100.00% (100.00%, 100.00%)	1.00

*TN*, true negative; *N*, negative; *TP*, true positive; *P*, positive; *Sensitivity*, dividing true positive by positive; *Specificity*, dividing true negative by negative

## Discussion

The study revealed that the integration of a real-time computer-aided polyp detection system during colonoscopy substantially improves efficacy by increasing adenoma and polyp detection rates, as well as enhancing accuracy in terms of sensitivity and specificity. Recent research has further established that artificial intelligence-assisted colonoscopy (AIC) serves as a safe and efficient screening tool, improving the detection rates of colorectal cancer adenomas and polyps in adults and facilitating early diagnosis of colorectal cancer when compared to conventional colonoscopy [18, 19].

Colorectal cancer screening methods are effective but have limitations, and many eligible individuals are unscreened. This paper reviews current and new screening techniques and suggests ways to overcome these limitations. While new strategies could revolutionize prevention, significant improvements can be made by optimizing current colonoscopy methods. Colonoscopy diagnostics have a rich history [33].

Over the past decades, alternative methods such as CT colonography, colon capsule endoscopy, and AI-assisted detection have been explored to address the limitations of traditional gastrointestinal endoscopy [34, 35]. While auxiliary inspection techniques can enhance detection rates, the sensitivity of white light colonoscopy often fails to identify invasive flat or depressed lesions, which may progress to larger, advanced tumors. Fluorescence molecular imaging addresses this shortcoming by significantly improving the detection rate of precancerous lesions [36]. In a previous study [35], it was demonstrated that AI-assisted colonoscopy enhanced overall ADR, advanced ADR, and ADR among both expert and non-expert endoscopists. In addition, the implementation of a novel AI detection system resulted in a significantly higher number of adenomas detected per colonoscopy compared to conventional high-definition colonoscopy, without extending the colonoscopy withdrawal time. This finding supports the use of AI-assisted colonoscopy to enhance the quality of colonoscopy in a large, prospective, multicenter, randomized clinical trial [37].

As emerging technologies continue to develop, computer-aided science has been increasingly applied across various fields, particularly in medicine, to enhance patient quality of life. Gastrointestinal endoscopy, with its long-standing history in detecting, diagnosing, and treating colorectal lesions [33, 38], remains the most suitable test for high-risk individuals or as a follow-up procedure after a positive initial test [39]. Despite the current advancements in gastroenterology, there are perspectives that express reservations about the technological future of endoscopy. To optimize gastrointestinal endoscopy for reducing the risk of colorectal cancer, it is essential to refine our strategies to enhance the detection of adenomas and polyps [40].

Notably, our findings indicate an inverse relationship between the adenoma detection rate (ADR) and the risk of colorectal cancer at any stage. Furthermore, increasing polyp detection rates can significantly lower the incidence of colorectal cancer [21, 41, 42]. Consequently, we employed ADR and polyp detection rate (PDR) as key indicators in numerous studies to assess the effectiveness of a real-time computer-aided polyp detection system during colonoscopy [43]. Previous research has established a strong correlation between polyps and the onset and progression of colorectal cancer [23]. Therefore, to improve early-stage colorectal cancer detection, our objective is to elevate polyp detection rates, given that colorectal cancer is a genetic disease originating from precursor colon lesions or polyps that evolve through various tumorigenesis pathways [44]. Artificial intelligence (AI) has the potential to enhance polyp detection; however, numerous uncertainties persist. Despite minor variations among different AI algorithms, the overall efficacy of AI in polyp detection is promising, as evidenced by clinical trials [45]. Various strategies have been identified to improve the polyp detection rate [46, 47]. Nevertheless, a more critical issue lies in the limitations of algorithmic procedures. While CADe has been shown to increase adenoma detection rates in randomized trials, it still faces significant challenges, particularly in identifying certain pathological types of lesions. For instance, the first commercially available CADe program in the United States, GI Genius, demonstrates suboptimal performance in detecting large, flat lesions. Similarly, although the new Medtronic CADe program, ColonPRO, incorporates advanced AI capabilities, preliminary evaluations indicate that it continues to struggle with effectively detecting certain lesions, especially after excluding specific patient groups. Certain lesions necessitate modifications in colonoscopy procedures to elicit signals, whereas others may not generate any signals. The efficacy of CADe is contingent upon the training of the algorithm. If the algorithm is primarily trained to identify small lesions, it may fail to detect large, flat lesions, potentially increasing the ADR but not effectively preventing colorectal cancer. Thus, it is imperative to balance the algorithm to detect all types of lesions for effective colorectal cancer prevention [48]. A meta-analysis indicates that AI-assisted colonoscopy can substantially enhance the adenoma detection rate; however, regardless of the endoscopists’ experience, system type, or medical environment, it does not improve the detection rate of sessile serrated lesions. This suggests inherent limitations within the artificial intelligence algorithm, aligning with our statistical findings, where external factors were not statistically significant [49].

In this prospective trial, we analyze and compare the adenoma detection rate (ADR) and polyp detection rate (PDR) between standard colonoscopy and computer-aided colonoscopy to evaluate the efficacy and monitor the safety of the system during the procedure. The study aims to assess the accuracy and safety of the computer-assisted diagnostic software for detecting intestinal polyps via gastrointestinal endoscopy, building upon previous research involving colonoscopy screening with fecal occult blood tests [50]. Our study employs the polyp per colonoscopy (PPC) as the primary outcome measure, a metric routinely recorded during gastrointestinal polyp endoscopy, thereby enhancing the feasibility of data collection. Preliminary data indicate that software-assisted gastrointestinal polyp endoscopy can significantly increase PPC. This trial is informed by prior exploratory clinical research, providing valuable experience in project design, training, implementation, and statistical analysis, which enhances the probability of success.

Researchers have employed various approaches to investigate the potential of artificial intelligence (AI) in enhancing the detection of adenomas and polyps, which holds significant implications for the prevention of colorectal cancer [51]. The methodology of our study parallels that of a randomized trial, which prospectively assessed the use of a computer-aided detection device across five academic and community centers by gastroenterologists certified by the US board [52]. In both studies, participants were randomly assigned to two groups to evaluate whether AI assistance could improve the detection rates of adenomas and polyps. However, while the previous study assessed safety based on the severity of adverse events and their relationship to the intervention, our study evaluated safety by examining the rates of bleeding and perforation. In addition, we drew insights from a prior trial that explored AI-assisted adenoma detection [53].

While the results align with the hypothesis, the study has several flaws, notably the lack of a double-blind design. This trial examines PPC differences between experimental and control groups, primarily influenced by the number of polyps and endoscopists’ detection abilities. Extremely low or high polyp numbers or a very low missed diagnosis rate could lead to negative outcomes. However, the study’s population, individuals consecutively needing endoscopy, should have a moderate polyp count. In addition, previous research indicates even experienced physicians have some missed diagnoses, reducing the likelihood of trial failure.

These findings suggest a promising avenue for the prevention of colorectal cancer by emphasizing the advancement of artificial intelligence technologies. AI holds substantial potential in the creation of innovative screening strategies designed to enhance the detection of adenomas and polyps, thereby contributing to an improved quality of life. The application of AI across diverse medical domains has yielded favorable outcomes, significantly enhancing diagnostic and detection rates, and consequently improving overall quality of life [54].

Future research should consider employing double-blind designs to more accurately evaluate the specific role of the system in enhancing adenoma detection rates, as the observed behavior may influence these rates in the experimental group. Given that baseline adenoma and polyp detection rates may be affected by regional characteristics, the study’s findings may not be applicable to regions with higher baseline adenoma detection rates globally. Further investigation is required to assess the system’s adaptability and effectiveness in such regions. Research should focus on directions such as improving processing speed while maintaining high accuracy to meet the demands of real-time examinations. In addition, large-scale, multicenter randomized controlled trials are necessary to validate the effectiveness of artificial intelligence systems in clinical settings. Customizing models based on the specific characteristics of different hospitals and equipment could enhance treatment outcomes.

In conclusion, this study demonstrated the efficacy and accuracy of the software in real-time colonoscopy and identified the sensitivity and specificity of the system.

## Data Availability

The authors would provide the raw data that support the conclusions of this article, without any undue restrictions.
